# Cause of Epidemics

**Published:** 1863-02

**Authors:** 


*Cause of Epidemics.*—Dr. JAS. GALBRAITH suggests that as decomposing organic matter upon the surface of the earth may generate gases productive of endemic or sporadic disease, so may gases arising from the underlying formations become the exciting cause of various epidemics, or result in change of the general constitution of the air, varying the type of existing diseases. Volcanic action, earthquakes, etc., may liberate noxious gases, not only where they are immediately observed, but at great distances, disturbing the crust of the earth and contaminating the atmosphere, the waters of springs and rivers, etc.,—thus influencing powerfully the conditions of human life.

